# Incidence of HIV-positive admission and inpatient mortality in Malawi (2012-2019): a population cohort study

**DOI:** 10.1097/QAD.0000000000003006

**Published:** 2021-11-01

**Authors:** Rachael M Burke, Marc Y R Henrion, Jane Mallewa, Leo Masamba, Thokozani Kalua, McEwan Khundi, Ankur Gupta-Wright, Jamie Rylance, Stephen B Gordon, Clemens Masesa, Elizabeth L Corbett, Henry C Mwandumba, Peter Macpherson

**Affiliations:** (1) Clinical Research Department, Faculty of Infectious and Tropical Disease, London School of Hygiene and Tropical Medicine; (2) Malawi Liverpool Wellcome Clinical Research Programme, University of Malawi College of Medicine; (3) Department of Clinical Sciences and International Public Health, Liverpool School of Tropical Medicine; (4) Department of Medicine, Queen Elizabeth Central Hospital, Blantyre, Malawi; (5) Department of HIV and AIDS, Ministry of Health, Lilongwe; (6) Faculty of Epidemiology and Population Health, London School of Hygiene and Tropical Medicine

**Keywords:** HIV, epidemiology, hospital, mortality, ART, temporal trends

## Abstract

**Objective:**

To investigate trends in population incidence of HIV positive hospital admission and risk of in-hospital death among adults living with HIV between 2012 and 2019 in Blantyre, Malawi.

**Design:**

Population cohort study using an existing electronic health information system (‘SPINE’) at Queen Elizabeth Central Hospital and Blantyre census data.

**Methods:**

We used multiple imputation and negative binomial regression to estimate population age- and sex-specific admission rates over time.We used a log-binomial model to investigate trends in risk of in-hospital death.

**Results:**

Of 32,814 adult medical admissions during Q4.2012-Q3.2019, HIV status was recorded for 75.6%. HIV-positive admissions decreased substantially between 2012 and 2019. After imputation for missing data, HIV positive admissions were highest in Q3.2013 (173 per 100,000 adult Blantyre residents) and lowest in Q3.2019 (53 per 100,000 residents). An estimated 10,818 fewer than expected people living with HIV (PLHIV) (95%CI 10,068-11,568) were admitted during 2012-2019 compared to the counterfactual situation where admission rates stayed the same throughout this period. Absolute reductions were greatest for women aged 25-34 years (2,264 fewer HIV-positive admissions, 95%CI 2,002-2,526). In-hospital mortality for PLHIV was 23.5%, with no significant change over time in any age-sex group, and no association with ART use at admission.

**Conclusions:**

Rates of admission for adult PLHIV decreased substantially, likely due to large increases in community provision of HIV diagnosis, treatment and care. However, HIV-positive in-hospital deaths remain unacceptably high, despite improvements in ART coverage. A concerted research and implementation agenda is urgently needed to reduce inpatient deaths among PLHIV.

## Introduction

The Joint United Nations Programme on HIV/AIDS (UNAIDS), national country HIV programmes and many other actors in the HIV community share a common goal to end AIDS as a public health problem by 2030. In sub-Saharan Africa, great progress has been made towards goals of achieving 95% of people living with HIV knowing their status, 95% of those who know their status to be taking antiretrovial therapy (ART), and 95% of those of those taking ART to have undetectable HIV viral loads. Malawi is one of countries worst affected by the HIV epidemic, with estimated adult HIV prevalence in 2019 of 8.9% nationwide and 17.7% in Blantyre City.(1) In the past two decades the Malawi national HIV programme has made excellent progress in providing HIV testing, ART and other HIV care services; in 2019, 90% of all PLHIV in Malawi knew their HIV status, 88% of those who knew their status were taking ART and 92% of those on ART were virally suppressed.(2)

Despite increasing population ART coverage, the number of PLHIV becoming unwell and attending hospital has remained high in several countries in Southern and Eastern Africa. For example, 60% of hospital admissions to a general hospital in South Africa were related to HIV in 2012-13, despite widespread ART availability in the community at that time.(3) Similarly, 50% and 42% of admissions to hospital in Lilongwe, Malawi between 2011 and 2012 and Kweneng East District, Botswana between 2015 and 2016, respectively, were related to HIV.(4, 5) Another study found that 83% and 97% of PLHIV admitted to hospitals in Kenya and DRC respectively had advanced immunosuppression (CD4 <200 cells/mm^3^).(6) In general, hospital epidemiological data related to HIV in Southern and Eastern Africa is sparse. In Johannesburg, South Africa, 39% of people initiating ART in 2017 had CD4 <200, indicating that advanced HIV remains a persistent challenge.(7–9)

We used routine hospital data and city census data to investigate changes in HIV-positive hospital admissions to adult medical wards over time in Blantyre, Malawi, where there is only one public hospital serving the population, acting as both District General Hospital and a tertiary referral hospital. The primary objective was to assess time trends in the incidence (i.e. number of hospital admissions per 100,000 population) of HIV-positive hospital admission for Blantyre residents between 2012 and 2019. The secondary objective was to investigate whether hospital admission outcomes (died vs. discharged from hospital alive) for people living with HIV (PLHIV) have changed over time.

## Methods

### Setting

Blantyre District contains the second largest city in Malawi (Blantyre City) and it’s surrounding periurban/rural area. At the 2018 census, Blantyre District had a population of approximately 1.2 million people with a median age 17 years.(10) One main government hospital (Queen Elizabeth Central Hospital, QECH) provides free secondary and tertiary care to the population of Blantyre, including inpatient medical care. There are some smaller private (including private-not-for-profit) hospitals accessed by a small sub-set of the population who can afford the fees, but the vast majority of people living in Blantyre rely on QECH exclusively for inpatient care. QECH provides a range of general medical services, HIV testing (provider-initiated testing and counselling [PITC]) and ART. QECH has 120 general adult medical beds and this capacity hasn’t substantially changed between 2012 and present.

### Population and data sources

Since late 2009 adult medical admissions to QECH have been recorded in in an electronic surveillance system (Surveillance Programme of IN-patients and Epidemiology [SPINE]) by data clerks working on both of the medical admissions wards.(11) For all patients admitted to the wards, data clerks recorded: sex, age, neighbourhood of residence, date of admission, HIV status, ART status and outcome (discharge from hospital alive vs. died prior to discharge). Individual patients are not linked over time, and results of CD4 cell counts or HIV viral load tests are not recorded. ART status was ascertained from medical notes or a patient-held record (“health passport ”) during admission. Quality is assured by reconciling admissions with government paper ledgers, nurses’ paper records and data clerks physically walking around bed spaces each morning. There was some interruption to SPINE data collection in 2011–2012, so we included medical admissions recorded by SPINE from October 2012 to September 2019. We removed duplicate records, records for people under 15 years old and records for in patients who reported residing outside of Blantyre. We assumed that those with missing location data lived in Blantyre.

The government of Malawi conducted population censuses in 2008 and 2018. Mid-quarter population estimates for Blantyre (combining “Blantyre urban ” and “Blantyre rural ” areas) for each quarter between October 2012 and September 2019 were calculated by linear interpolation and extrapolation, by 10-year age group and sex.

### Statistical analysis

Characteristics of patients admitted to QECH medical wards were summarised using percentages, and compared to interpolated Blantyre census data. Where data on HIV status, ART and outcome were missing in SPINE, we used multiple imputation by chained equations (using the ‘mice’ package in R) with predictive mean matching to impute missing data.(12) Variables used for imputation were HIV status, age group, quarter-year, sex and outcome. Missing ART status for the small number of people who reported being HIV positive was also imputed based on the above variables. Since ART status missingness is conditional on HIV status missingness, we did not impute ART status for people who had missing or unknown HIV status in SPINE. For the secondary outcome assessing assosciations with in-hospital death, we assumed that everyone who was HIV positive (based in imputation) but had an unknown or missing HIV status in SPINE was not taking ART – this was not relevant for the primary outcome of incidence of admission. We imputed 25 datasets (reflecting the ˜25% missingness of ART status), and combined model outputs across all 25 datasets using Rubin’s rules.(13–15) Sensitivity analyses were performed by conducting complete case analysis; for HIV-related admission incidence analysis, complete case analysis is equivalent to assuming all participants with unknown HIV status were HIV-negative.

We estimated the incidence of HIV-positive and HIV-negative admission to hospital among Blantyre residents per quarter-year between Q4.2012 and Q3.2019 overall, and separately for each age group-sex-quarter strata. To investigate trends in admission over time, we fitted a negative binomial regression model (because the data were overdispersed) with interactions between age group, sex and quarter, and a natural cubic spline term with three knots for annual quarter. Age group and sex were included as interaction variables in the models *a priori* because there are sex and age-group specific differences in HIV incidence, prevalence, and access to testing and ART services. We performed sensitivity analyses using the Poisson and gamma response distributions, and separately without spline terms.

To quantify the magnitude of change in admissions over the study period overall, and for each age group-sex strata, we calculated the expected number of admissions under the counterfactual condition where the incidence of HIV positive admission remained constant as the model predicted for Q4.2012 (ie. the first quarter of observation) over the entire study period, and subtracted from the model-predicted number of admissions. Confidence intervals were estimated using parametric bootstrap resampling.

Temporal trends in the risk of inpatient death were analysed using a generalised linear model with log-binomial link function to approximate risk of death. Age group and sex were included as interaction variables *a priori*. We investigated whether adding ART use at admission (including ART used as an interaction variable with age, sex and quarter-year) improved model fit using Akaike information criteria.

### Ethical approval, funding and data sharing

Use of anonymous electronic data (from SPINE project) was approved by QECH hospital research committee. Individual patient consent for anonymised secondary analysis was not sought.

All code for analyses, Blantyre census dataset, datapoints from figures and a ‘synthetic’ (i.e. artificial data that mimics properties of real data) dataset for hospital admissions are available online at https://rachaelmburke.github.io/hivhospital/. Synthetic data was created using synthpop package.(16) Further details including how to access real data are included in data sharing statement.

SPINE received funding from Wellcome Core Grant to the Malawi-Liverpool-Wellcome Trust (reference 206545). RMB, ELC and PM are funded by Wellcome (203905/Z/16/Z, 200901/Z/16/Z, and 206575/Z/17/Z, respectively).

## Results

During the 28 quarters between October 2012 and September 2019, there were 32,814 medical admissions to QECH among adults (age ≥15 years) who resided in Blantyre (median quarterly admissions 154 per 100,000 people). There were a further 5,511 people admitted to QECH who reported residing outside of Blantyre, and their data were excluded from this analysis. Fifty percent (16,408) of these were known to be HIV-positive, and in 24% (7,996) of admissions, HIV status was unknown (Table 1).

### Incidence of HIV related admission

The median number of known HIV-positive admissions (i.e. before imputation for missing HIV status) to QECH per quarter-year was 592 (80 per 100,000 Blantyre population). It was highest in Q3 2014 (767 known HIV-positive admissions, 110 per 100,000) and lowest in Q22019 (343 known HIV-positive admissions, 44 per 100,000) in Q2 2019. In contrast, known HIV-negative admissions were at their lowest towards the start of the study period, with 160 admissions (23 per 100,000 population) in Q3 2013 and highest in Q3.2019 with 482 admissions (61 per 100,000). The number of admissions with unknown or missing HIV status decreased throughout the study period, with a 695 admissions with HIV status missing or unknown in Q3 2017 (102 per 100,000) and 104 HIV unknown admissions in Q1 2017 (13 per 100,000). The proportion of people currently taking ART among known PLHIV admitted to hospital increased from 66% (363/550) in Q4 2012 to 94% in Q3 2019 (372/402); the denominator includes those who knew their HIV status prior to admission and those newly diagnosed in hospital, but not those who had missing or unknown HIV status recorded. Supplementary Figures 1A-C show HIV status, absolute number and population incidence of admissions over time. The adult Blantyre mid-year census population was 577,893 in 2008 and was 764,323 in 2018. The estimated population in February 2016 (i.e. the mid study period) was 722,377 (Supplementary Table 1B).

### Multiple imputation and modelling trends in incidence of HIV-related hospital admission

After using multiple imputation to impute HIV status for the 24% (7,996/32,814) of people where it was unknown, estimated true HIV-positive admissions were highest in Q3 2013 with 1169 admissions (173 per 100,000) and lowest in Q2 2019 with 417 admissions (53 per 100,000). If we assume that all those with missing or unknown HIV status in SPINE but who were HIV positive based on imputation were not taking ART, then ART coverage increased from 48% in Q4 2012 to 76% in Q3 2019.

**Figure 2:**
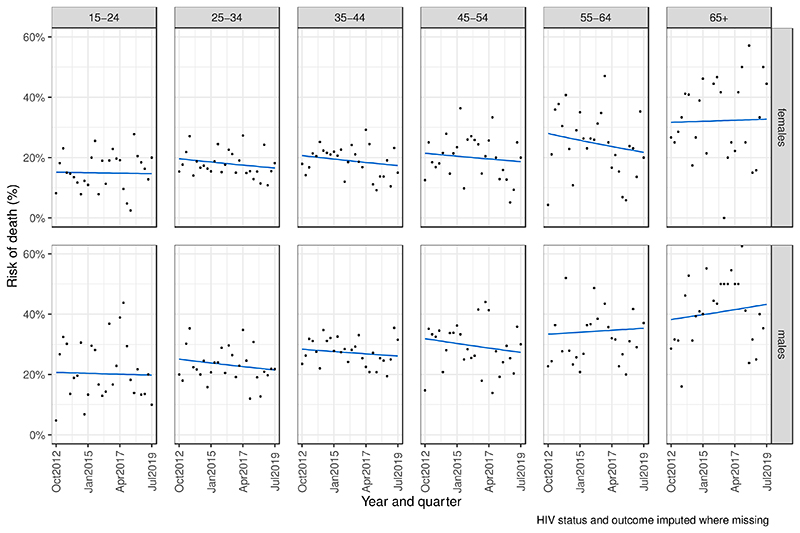
Risk of inpatient death among PLHIV if admitted to Queen Elizabeth Central Hospital, Malawi, Q3 2012 – Q3 2019. Log-binominal model.

Using regression modelling with parameters averaged across 25 multiply-imputed datasets, we estimate that the true number of HIV-positive hospital admissions between Q4 2012 and Q3 2019 (inclusive) was 21,170 (95% confidence interval [CI] 20,411–21,928). Between October 2012 and September 2019, the modelled trend of incidence of HIV-positive hospital admission in Blantyre decreased in all age and sex groups (Figure 2). In sensitivity analysis, this overall finding was robust to reclassification of missing HIV data (Table 2 and Supplementary Figures 2A and 2B), and to model specification (Supplementary Figures 3A-3D).

If the age group- and sex-specific incidence of HIV related hospital admissions had stayed the same throughout the period October 2012–September 2019 as it was in Q4 2012, then we would have expected to see 31,988 (95% CI 31,229–32,746) HIV-positive admissions, taking into account the increasing population of Blantyre. Therefore, we estimate that there were 10,818 (95% CI, 10,060–11,577) fewer HIV-positive admissions during this period than there would have been under counterfactual scenario where incidence of admission had remained constant during this period (Table 2). This is equivalent to 33.8% fewer HIV-positive admissions (95% CI 32.3% to 35.4%).

The greatest reductions in absolute numbers of admissions compared to expected number of admissions had there been no change in population incidence of admission were in women aged 25–34 years old and men aged 35–44 years old. The smallest magnitude of absolute decline in admissions were in men aged 55–64 years old and men age 65+ (Figure 2 and Supplementary Table 2).

These estimates were robust to reclassification of missing HIV status. If all admissions with missing HIV status were considered to be HIV-negative we estimate there would have been 3,854 (95% CI: 3,453 to 4,255) fewer HIV-positive admissions (equivalent to a 19.0% decrease), and if all admissions with unknown HIV status were considered HIV-positive, there would have been 13,865 (95% CI:13,050 to 14,681) fewer admissions (equivalent to a 36.2% decrease). In the sensitivity analysis scenario where all patients with missing HIV status were classified as HIV-negative, while overall HIV-positive admissions decreased, but there was no decrease in admissions among women aged 45 years or older, nor among men aged 65 years or older (Supplementary Table 2 and Supplementary Figure 2B).

During this period, the incidence of HIV negative hospital admissions stayed the same or increased in all age and sex groups (Figure 2) and increased substantially among those 65 years or older.

### Outcomes for PLHIV admitted to QECH

Overall, 18.5% (6,071/32,814) of adults admitted to QECH died during their admission, and a further 8% (2,687 / 32,814) had unknown outcome or missing outcome data. After multiple imputation, we estimate the proportion of adult medical inpatients who diedto be 20.3% overall and 23.5% among PLHIV (Table 3). Supplementary Table 3A and 3B show outcomes by age group and sex.

Risk of inpatient death did not change over the study period overall, or within any age-sex subgroups (Figure 2 and Supplementary Table 4). This finding was robust to sensitivity analyses for misclassification of HIV status and outcome (complete case analysis - Supplementary Figure 4). Reported ART use at admission did not affect the risk of in-hospital death and did not improve the model fit (Supplementary Figure 5); Akaike information criteria statistics were higher in models that included ART as a covariate in all 25 imputed datasets. Risk of death was higher for people living with HIV than people without HIV in all age and sex groups (Table 3, Supplementary Table 3, and Supplementary Figure 6).

## Discussion

We used electronic inpatient records and national census data to show that between 2012 and 2019, per capita rates of HIV-positive medical admissions in Blantyre, Malawi decreased substantially. There were an estimated 10,818 (95% CI: 10,068 to 11,568) fewer HIV-positive admissions to the single public hospital than would have been expected if admission rates had been unchanged from the last quarter of 2012. These data were adjusted for population growth, and excluded tertiary admissions referred from districts outside of Blantyre. The likely driver was ART scale-up, with substantial increases in community ART coverage during this time, consistent with the observed increase in the proportion of HIV-positive patients already on ART at the time of admission. Once admitted, however, mortality remained extremely high with 23.5% of PLHIV dying before discharge, no obvious improvements over time, and no benefits from being on ART at the time of admission. High in-patient mortality following medical admission in Africa is a critical issue that needs to be investigated and addressed urgently.

The substantial reduction in admission rates is an encouraging finding, and is congruent with other data which indicate that the proportion of people living with HIV in Blantyre who know their status, are on treatment, and are virally supressed and therefore not experiencing medical complications has increased considerably between 2012 and 2019,(2) a tremendous testament to the Malawian National HIV Programme. Alternative explanations for our findings are less likely. Queen Elizabeth Central Hospital is the single government hospital for the city, and care has remained free of charge and available to the population with no substantial changes or prolonged disruption to services during this time. Of note, this analysis ends in September 2019, before any COVID-19 related disruption. Incidence of HIV-negative hospital admissions stayed the same or increased in every age and sex group during this time, consistent with investments in health system strengthening and indicating that the decline in HIV positive admissions is not a data capture issue.

To put these results into context; estimated national adult HIV prevalence in Malawi was relatively static between 2012 and 2019, although AIDS deaths and new HIV infections fell, concurrent with rising coverage of ART.(17) There are limited subnational HIV estimates for Blantyre derived from NAOMI/Spectrum models, with estimates available for March 2016 and December 2019 only.(18,19) Similar to national estimates, Blantyre adult HIV prevalence was largely unchanged at 17.0% in March 2016 and 16.7% in December 2019. Blantyre ART coverage increased, from 60.1% in March 2016 to 73.6% in December 2019; which is similar to the observed ART coverage in our analysis. Nationally, the peak of AIDS related deaths in Malawi was in 2004 with 71,000 deaths, several years before the SPINE database was commenced. Between 2012 and 2019 (i.e. the dates of this analysis) national HIV related deaths declined from 24,000 annually to 13,000, with steeper declines at the start of this time period. There are no subnational estimates for deaths. As an approximate estimate — assuming that the proportion of HIV related deaths in Blantyre compared to the rest of Malawi is the same as the proportion of people living with HIV in Blantyre compared to the rest of Malawi — in 2018, between one quarter and one third of all Blantyre HIV related deaths occurred in QECH and were captured in this analysis. Our hospital observations are consistent with the modelled national and subnational trends; this analysis provides a further demonstration from empiric longitutinal data (rather than modelled data) of the impact of ART on the HIV epidemic in Malawi.

Once admitted to medical wards the risk of in-hospital death remained high and unchanged throughout the seven-year study period, being 23.5% for HIV-positive medial inpatients and 14.5% for HIV-negative inpatients, once missing HIV status and outcomes were imputed. Although ART coverage among PLHIV admitted to hospital increased substantially between 2012-19 (commensurate with increasing population ART coverage), ART use at admission did not alter risk of death. The impact of virological failure in this cohort can only be inferred, as HIV viral load measurement on admission is not currently supported by the routine medical services, and data on HIV viral loads are not routinely captured. Studies that have measured HIV virologic failure among people in hospital have shown similarly high mortality and high levels of proven HIV virologic failure among people admitted to hospital. In the STAMP trial in 2015 to 2017 in Zomba Central Hospital, Malawi (in a nearby district to Blantyre), 32% of all PLHIV admitted to hospital had confirmed HIV virologic failure and this was associated with increased risk of death.(20) Other African studies report a high prevalence of HIV virologic failure among PLHIV admitted to hospital; 63% and 62% in Kenya and Democratic Republic of Congo, respectively.(6) In a predictive model developed using STAMP data and validated on cohorts from another multi-centre trial and a cohort in Kenya, use of ART at admission to hospital was associated with increased risk of death by two months from admission.(21) In the present analysis, use of ART made no difference to risk of in-hospital death.

At the start of the study period, slightly less than half of all people with HIV were taking ART. It is possible that, for those that survived the acute illness that precipitated admission, effective ART could be started and outcomes may be relatively favourable. By the end of the study period three quarters of HIV positive peole admitted to hospital were taking ART. If a substantial proportion of those on ART had HIV virologic failure and were not switched to effective ART, then they may be discharged with their acute illness treated, but the underlying immunosuppression that precipitated the illness unresolved. At present, WHO guidelines for managing confirmed or suspected HIV virologic failure do not distinguish between stable ambulatory outpatients and unwell patients admitted to hospital, and recommend enhanced adherence counselling following identification of an elevated HIV viral load.(22) There are scant data to address this issue or provide guidance as which groups of people require urgent ART switch and in which groups of people adherence counselling and repeat viral load may be appropriate.

In a meta-analysis of PLHIV admitted to hospital, AIDS-related conditions (including tuberculosis and cryptococcal meningitis) and severe bacterial infections were the most common causes of admission and death,(23) consistent with previous data from QECH about cause of admission,(11) and suggesting that for most people living with HIV their HIV status is not incidental to the reason for hospital admission. Two trials have shown that urine-based TB diagnostics reduce deaths of PLHIV in hospital,(20,24) and several trials have shown effectiveness of newer antifungal treatments for cryptococcal meningitis.(25,26) However, there are no trials of pragmatic management protocols (which might include a package of diagnostics), or of interventions to optimise management of virological failure among people in hospital. In the era of universal ART coverage, PLHIV admitted to hospital should be managed with great urgency, given their high risk of imminent death, and we urge more trials to produce evidence-based pragmatic management protocols similar to those recently developed for patients with low CD4 counts.(27,28)

There are some limitations to this work. We do not have information on cause of admission or cause of death for those who died. Similarly, we do not have information on HIV viral loads or CD4 counts, to be able to measure prevalence of advanced HIV or HIV virologic failure directly. There was no follow up beyond length of hospital stay to ascertain mortality in the immediate period after admission. Malawi has very recently switched first-line ART to a dolutegravir-based regimen, away from reliance on non-nucleoside reverse transciptase inhibitors (NNRTIs), including switching those who are stable on NNTRI-containing ART regimens; the switch occurred in 2019, but this is too early to observe if this will have causes any change in HIV related hospital admissions. QECH has a large outpatient ART service, so it is possible that people who were taking ART were more likely than those not on ART to be admitted to hospital (either due to emergency referral from ART clinic or from being familiar with services available at the hospital). This might mean that the proportion of inpatients taking ART is higher than the proportion of all people who are sick (but don’t access QECH hospital care) who are taking ART.

In conclusion, the incidence of HIV-positive hospital admission in Blantyre has substantially reduced in the seven years between Q4.2012 to Q3.2019, in keeping with impressive gains in coverage of HIV testing, treatment and care in Malawi during this period. However, PLHIV who were admitted to hospital continued to experience extremely high in-hospital mortality that did not change throughout this period. This suggests that advanced HIV and HIV-related complications remain persistent clinical and public health challenges, even as large improvements are made in providing HIV testing and care services to the majority of community members in Malawi. Interventions to reduce deaths in PLHIV following admission to hospital, including prompt management of HIV virologic failure in unwell and unstable patients, are an urgent research priority.

## Supplementary Material

Supplementary appendix

## Figures and Tables

**Figure 1:**
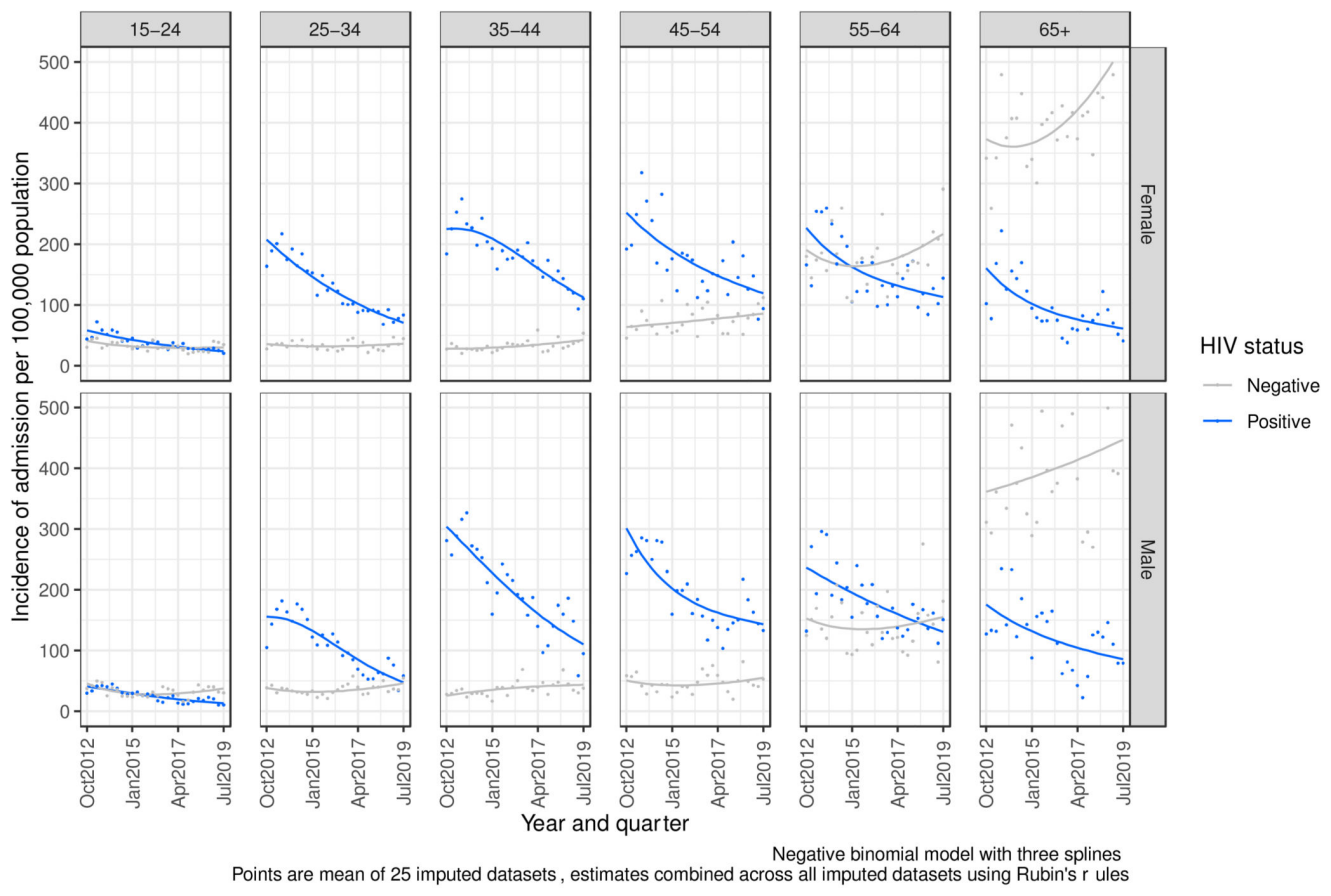
Population incidence of hospital admission to medical wards QECH by HIV status Q3 2012 – Q3 2019.

**Table 1:** Characteristics of adult medical admissions to Queen Elizabeth Central Hospital, Malawi, Q4 2012 – Q3 2019, and population demographics of Blantyre in Feb 2016 (midpoint Q3 2012 – Q3 2019)

	Adult medical admissions Oct 2012 to Sept 2019 (N=32,8I4)	Blantyre population estimates Feb 2016 (N=722,377)
**Age (years)**		
15-24	4,808 (14.7%)	270,260 (37.4%)
25-34	8,404 (25.6%)	197,589 (27.4%)
35-44	8,161 (24.9%)	131,376 (18.2%)
45-54	4,074 (12.4%)	60,267 (8.3%)
55-64	2,968 (9.0%)	32,416 (4.5%)
65+	4,399 (13.4%)	30,469 (4.2%)
**Sex**		
Females	16,618 (50.6%)	361,988 (50.1%)
Males	16,196 (49.4%)	360,389 (49.9%)
**HIV status**		
Negative	8,410 (25.6%)	
Positive	16,408 (50.0%)	
Missing or unknown	7,996 (24.4%)	
**ART status (HIV positive only)**		
Currently taking ART	13,074 (79.7%)	
Not currently taking ART	3,050 (18.6%)	
Missing or unknown	284 (1.7%)	
**Outcome from hospital admission**		
Alive	24,056 (73.3%)	
Dead	6,071 (18.5%)	
Missing or unknown	2,687 (8.2%)	

**Table 2:** Estimates of magnitude of reduction of HIV-related admissions

	Model predicted HIV-related admissions (Q4. 2012 – Q3. 2019), 95% confidence interval)			
Scenario	Number of HIV-related admissions predicted if incidence was the same throughout period as it was in Q4.2012 (A)	Estimated number of HIV-related admissions from regression model (B)	Absolute number fewer HIV-related admissions (A-B) *	Relative percentage decline in HIV related admissions (A-B / A) *
HIV status imputed when missing	31,988 (31,268 to 32,708)	21,170 (21,109 to 21,230)	10,818 (10,093 to 11,544)	33.8% (32.3 to 35.4%)
All HIV unknown / missing positive	38,270 (37,457 to 39,082)	24,404 (24,344 to 24,465)	13,865 (13,050 to 14,681)	36.2% (34.8 to 37.6%)
All HIV unknown / missing negative	20,262 (19,863 to 20,660)	16,408 (16,372 to 16,443)	3,854 (3,453 to 4,255)	19% (17.3 to 20.7%)

* Compared to counterfactual if admission incidence had stayed the same as it was in Q4.2012. 95% confidence intervals estimated through parametric bootstrapping of 25 multiply-imputed datasets

**Table 3:** Outcome of hospital admission (dead or discharged alive) by HIV and ART status

	Alive	Dead	Outcome missing	Overall
**Data without imputation**				
HIV negative	6767 (80.5%)	952 (11.3%)	691 (8.2%)	8410 (100%)
HIV positive (overall)	11387 (69.4%)	3276 (20.0%)	1685 (10.3%)	16408 (100%)
HIV positive, ART status unknown	177 (62.3%)	53 (18.7%)	54 (19.0%)	284 (100%)
HIV positive, not on ART	2200 (72.1%)	558 (18.3%)	292 (9.6%)	3050 (100%)
HIV positive, on ART	9070 (69.4%)	2665 (20.4%)	1339 (10.2%)	13074 (100%)
HIV status unknown or missing	5842 (73.1%)	1843 (23.0%)	311 (3.9%)	7996 (100%)
**TOTAL**	24056 (73.3%)	6071 (18.5%)	2687 (8.2%)	32814 (100%)
**Data with imputation (mean of 25** **imputations)**				
HIV negative	9935 (85.4%)	1701 (14.6%)	-	11636 (100%)
HIV positive (overall)	16249 (76.7%)	4929 (23.3%)	-	21178 (100%)
HIV positive, no ART status as HIV status imputed (likely no ART)	3495 (73.3%)	1275 (26.7%)	^-^	4770 (100%)
HIV positive, not on ART	2482 (79.5%)	640 (20.5%)	-	3122 (100%)
HIV positive, on ART	10272 (77.3%)	3014 (22.7%)	-	13286 (100%)
**TOTAL**	26184 (79.8%)	6630 (20.2%)	-	32814 (100%)

## Data Availability

All code for analysis and the Blantyre Census population denominator data is freely available online at https://rachaelmburke.github.io/hivhospital/. Unrestricted access to the SPINE dataset cannot be provided due to risk of reidentification of individuals. Instead a “synthetic” dataset is provided, created using ‘synthpop’ package in R statistical software. Synthetic data is artificial data that mimics some properties of the real data. It is intended to be used to be able to run and understand our code, but is not suitable for use in further analyses. The Malawi Liverpool Wellcome data department may be able to facilitate access to the real SPINE dataset and can be contacted on data@mlw.mw. Permission from QECH hospital is likely to be required. The first (rachael.burke@lshtm.ac.uk) and last author (peter.macpherson@lstmed.ac.uk) can also be contacted to enquire about how to access SPINE data. The dataset used for this analysis is anonymous and contains six variables (age, date of admission, sex, HIV status, ART status and outcome). Some of these combinations of variables include only one person and there is a theoretical risk of re-identification and disclosure of HIV status. Therefore it cannot be shared without restriction.
