# Supplementary material for: Incidence of HIV-positive admission and inpatient mortality in Malawi (2012-2019): a population cohort study
Source: AIDS. Author manuscript; Available in PMC 2021 Nov 15. (PMC7611991; doi:10.1097/QAD.0000000000003006)
Supplement: Supplementary appendix [file EMS129346-supplement-Supplementary_appendix.pdf]

**Supplementary Figure 1: Admissions by age, sex, HIV and quarter-year**

S. Figure 1A: Absolute numbers of admissions, stacked bar chart of crude data.

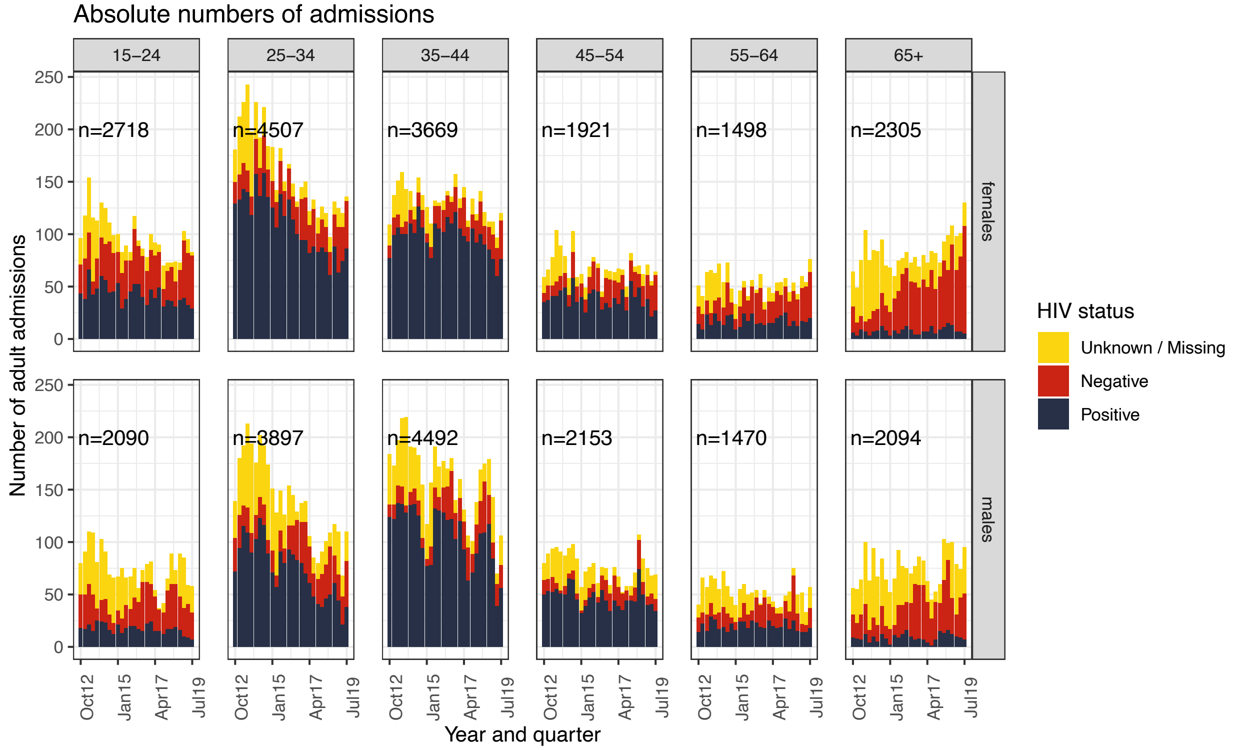


S. Figure 1B: Population level incidence of admissions, stacked bar chart of crude data.

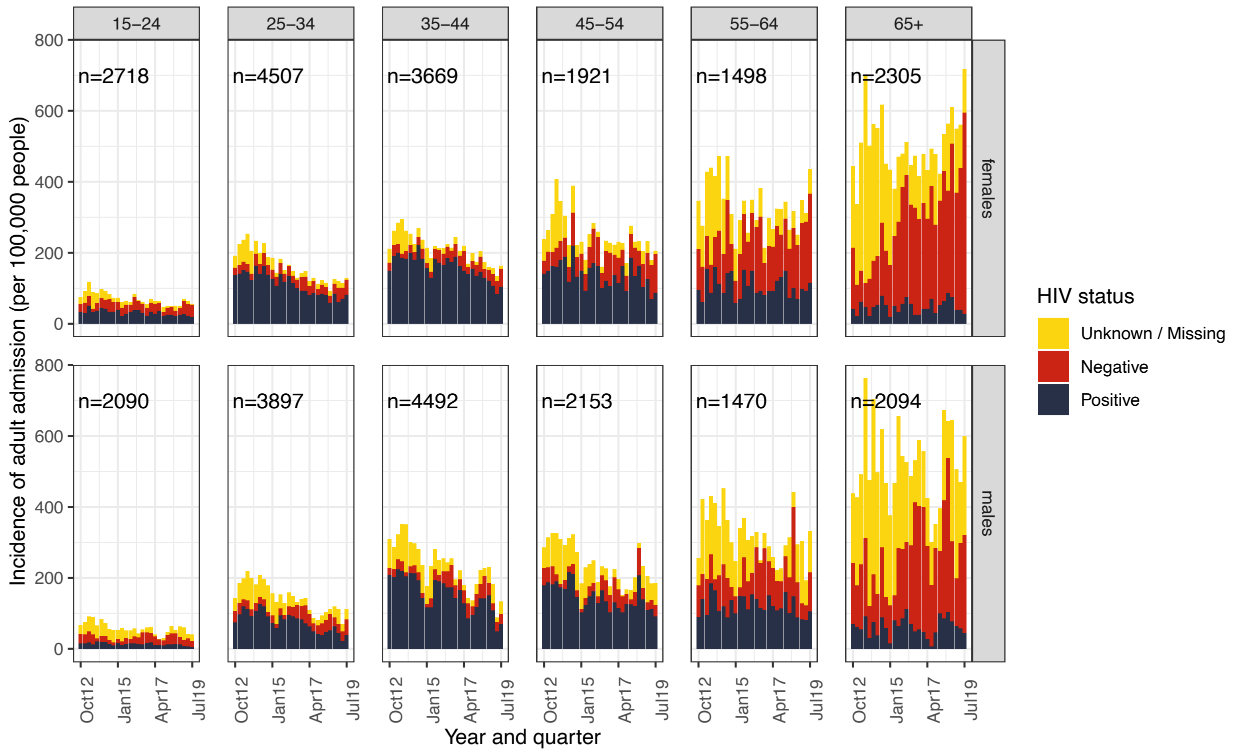

**Supplementary table 1: Blantyre census for 2008 and 2018**

S Table 1A: Measured population Blantyre (includes Blantyre City and Blantyre Rural administrative districts) at 2008 and 2018 census

| ageg10 | sex | 2008-04-01 | 2018-04-01 |
|---|---|---|---|
| 15-24 | females | 115573 | 146543 |
| 15-24 | males | 103290 | 138639 |
| 25-34 | females | 86762 | 104200 |
| 25-34 | males | 96314 | 97602 |
| 35-44 | females | 36968 | 69311 |
| 35-44 | males | 46176 | 76067 |
| 45-54 | females | 20676 | 29912 |
| 45-54 | males | 22000 | 35461 |
| 55-64 | females | 12915 | 16974 |
| 55-64 | males | 14529 | 16887 |
| 65+ | females | 11962 | 17441 |
| 65+ | males | 10728 | 15286 |

S Table 1B: Population Blantyre at start (Q4.2012) and end (Q3.2019) study period [calculated using linear interpolation and extrapolation]

| ageg10 | sex | 2012-10-01 | 2019-07-01 | change |
|---|---|---|---|---|
| 15-24 | females | 129510 | 150378 | 14% |
| 15-24 | males | 119197 | 143016 | 17% |
| 25-34 | females | 94609 | 106359 | 11% |
| 25-34 | males | 96894 | 97762 | 1% |
| 35-44 | females | 51523 | 73319 | 30% |
| 35-44 | males | 59627 | 79772 | 25% |
| 45-54 | females | 24832 | 31058 | 20% |
| 45-54 | males | 28058 | 37129 | 24% |
| 55-64 | females | 14743 | 17476 | 16% |
| 55-64 | males | 15591 | 17180 | 9% |
| 65+ | females | 14427 | 18121 | 20% |
| 65+ | males | 12778 | 15851 | 19% |

**Supplementary figure 2: Sensitvity analysis for incidence (imputation)**

S. Figure 2A: Everyone with HIV status unknown / missing is treated as if HIV negative

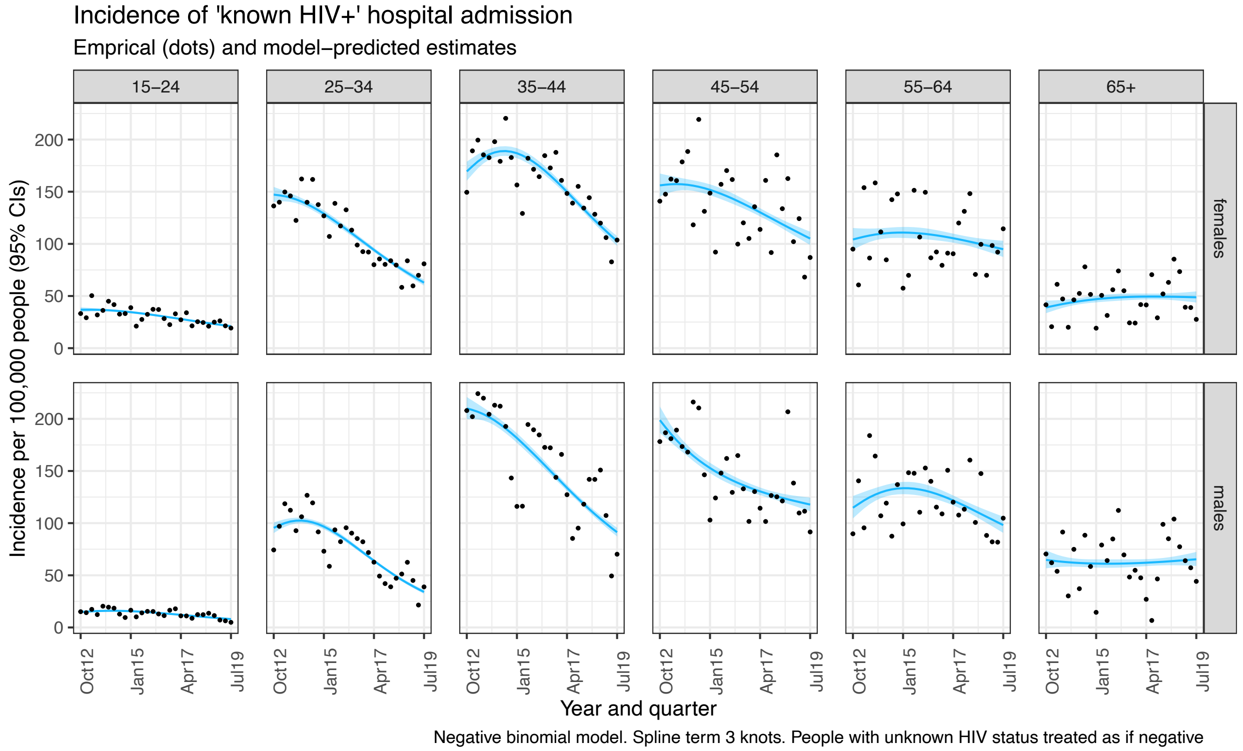


S. Figure 2B: Everyone with HIV status unknown / missing is treated as if HIV positive

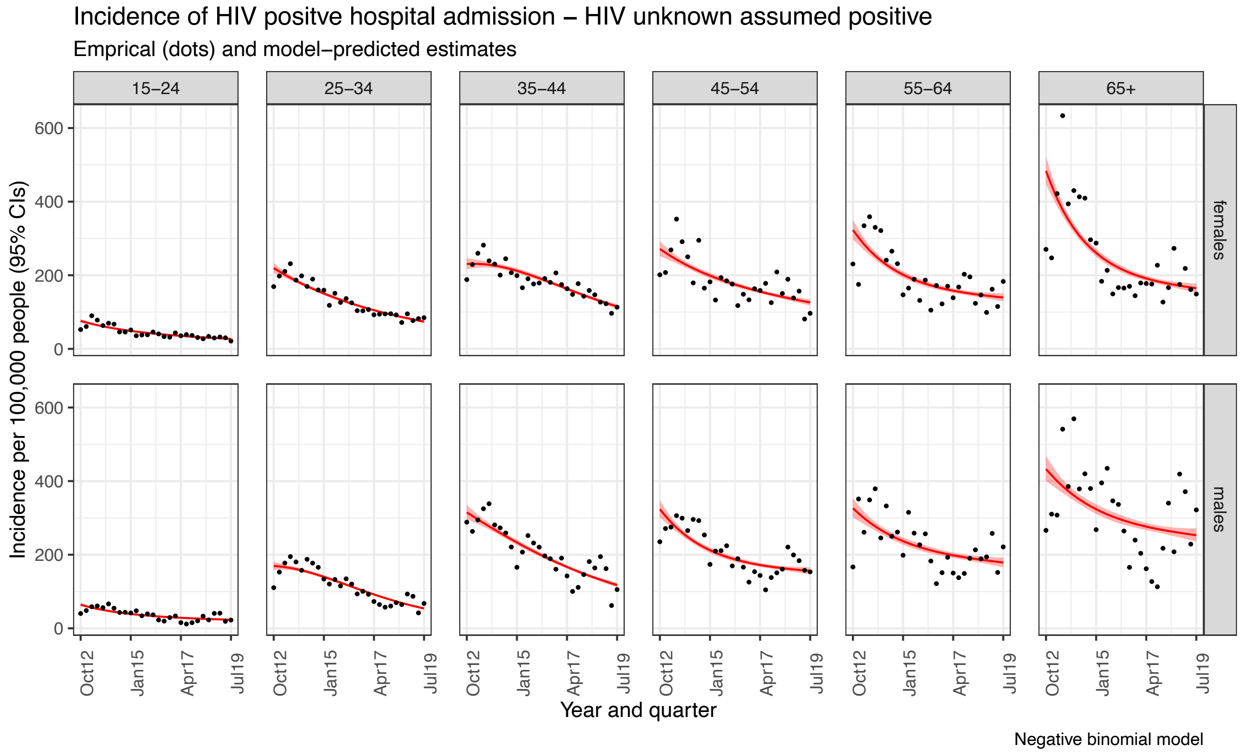

# Supplementary Table 2: Reduction in admission to QECH by age group and sex

| | | HIV status imputed when missing | | HIV unknowns assumed negative | | HIV unknowns assumed positive | |
|---|---|---|---|---|---|---|---|
| Age group | Sex | Absolute number | Relative decline | Absolute number | Relative decline | Absolute number | Relative decline |
| 15-24 | females | 815 (612 to 1019) | 0.356 (0.299 to 0.414) | 253 (164 to 342) | 0.175 (0.125 to 0.226) | 1248 (1049 to 1447) | 0.42 (0.381 to 0.459) |
| 15-24 | males | 599 (417 to 782) | 0.398 (0.325 to 0.472) | 66 (19 to 113) | 0.121 (0.045 to 0.198) | 1039 (879 to 1198) | 0.441 (0.403 to 0.479) |
| 25-34 | females | 2264 (1940 to 2589) | 0.388 (0.354 to 0.422) | 1078 (873 to 1283) | 0.26 (0.223 to 0.297) | 2474 (2096 to 2851) | 0.401 (0.364 to 0.437) |
| 25-34 | males | 1330 (1065 to 1595) | 0.314 (0.271 to 0.357) | 530 (392 to 669) | 0.204 (0.161 to 0.246) | 1489 (1194 to 1784) | 0.322 (0.279 to 0.366) |
| 35-44 | females | 844 (609 to 1080) | 0.215 (0.168 to 0.262) | 223 (61 to 385) | 0.075 (0.025 to 0.126) | 895 (632 to 1157) | 0.222 (0.171 to 0.273) |
| 35-44 | males | 2169 (1828 to 2509) | 0.366 (0.329 to 0.402) | 1121 (911 to 1331) | 0.274 (0.236 to 0.311) | 2255 (1877 to 2633) | 0.367 (0.328 to 0.406) |
| 45-54 | females | 635 (481 to 790) | 0.322 (0.269 to 0.375) | 149 (59 to 239) | 0.122 (0.058 to 0.187) | 713 (559 to 866) | 0.336 (0.287 to 0.384) |
| 45-54 | males | 1025 (836 to 1213) | 0.372 (0.329 to 0.415) | 497 (381 to 614) | 0.274 (0.227 to 0.321) | 1119 (909 to 1329) | 0.379 (0.334 to 0.423) |
| 55-64 | females | 337 (178 to 497) | 0.33 (0.226 to 0.435) | -6 (-55 to 43) | -0.013 (-0.119 to 0.094) | 595 (476 to 714) | 0.409 (0.36 to 0.457) |
| 55-64 | males | 267 (114 to 420) | 0.246 (0.139 to 0.353) | -30 (-82 to 21) | -0.058 (-0.162 to 0.047) | 461 (338 to 583) | 0.308 (0.251 to 0.364) |
| 65+ | females | 308 (156 to 459) | 0.421 (0.297 to 0.544) | -37 (-65 to -8) | -0.207 (-0.398 to -0.016) | 1081 (911 to 1252) | 0.491 (0.451 to 0.531) |
| 65+ | males | 225 (102 to 348) | 0.319 (0.2 to 0.438) | 9 (-26 to 43) | 0.033 (-0.096 to 0.162) | 498 (366 to 630) | 0.287 (0.233 to 0.341) |

**Supplementary figure 3A: Sensitivity analysis for incidence (choice of model)**

S. Figure 3A: Negative binomial distribution without spline terms

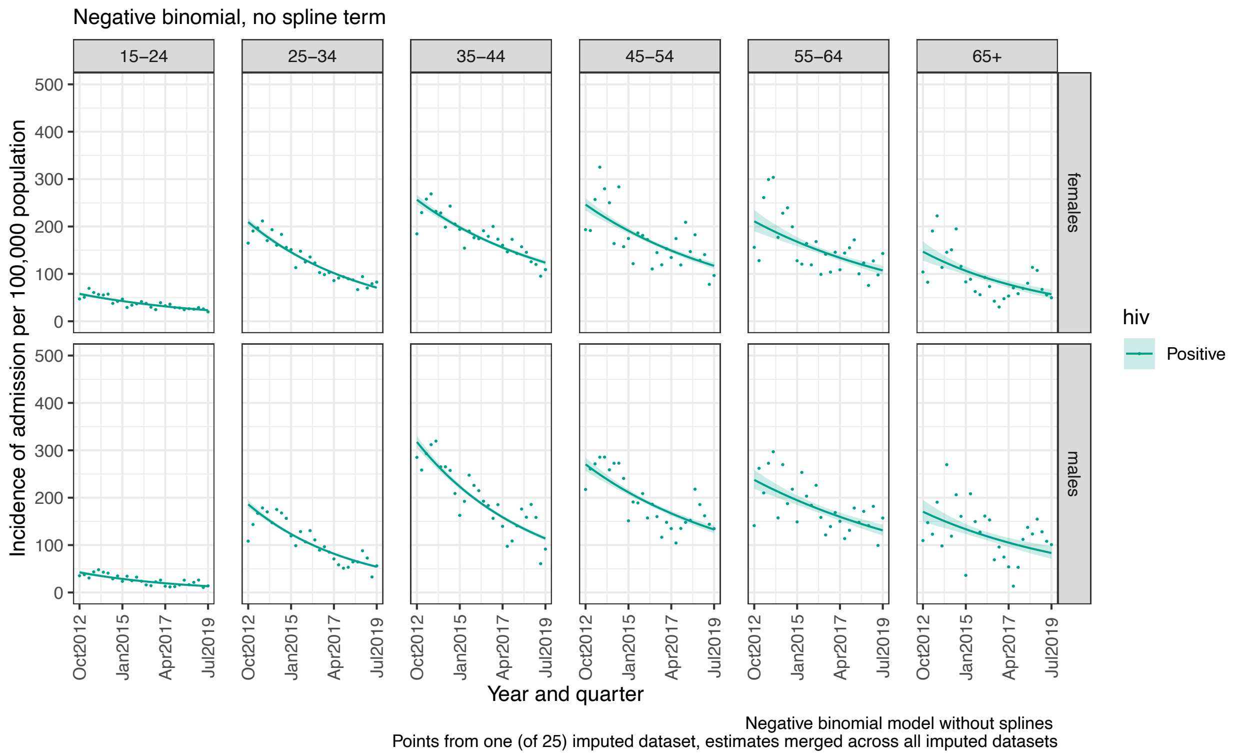


S. Figure 3B: Negative binomial distribution with 5 knots in spline

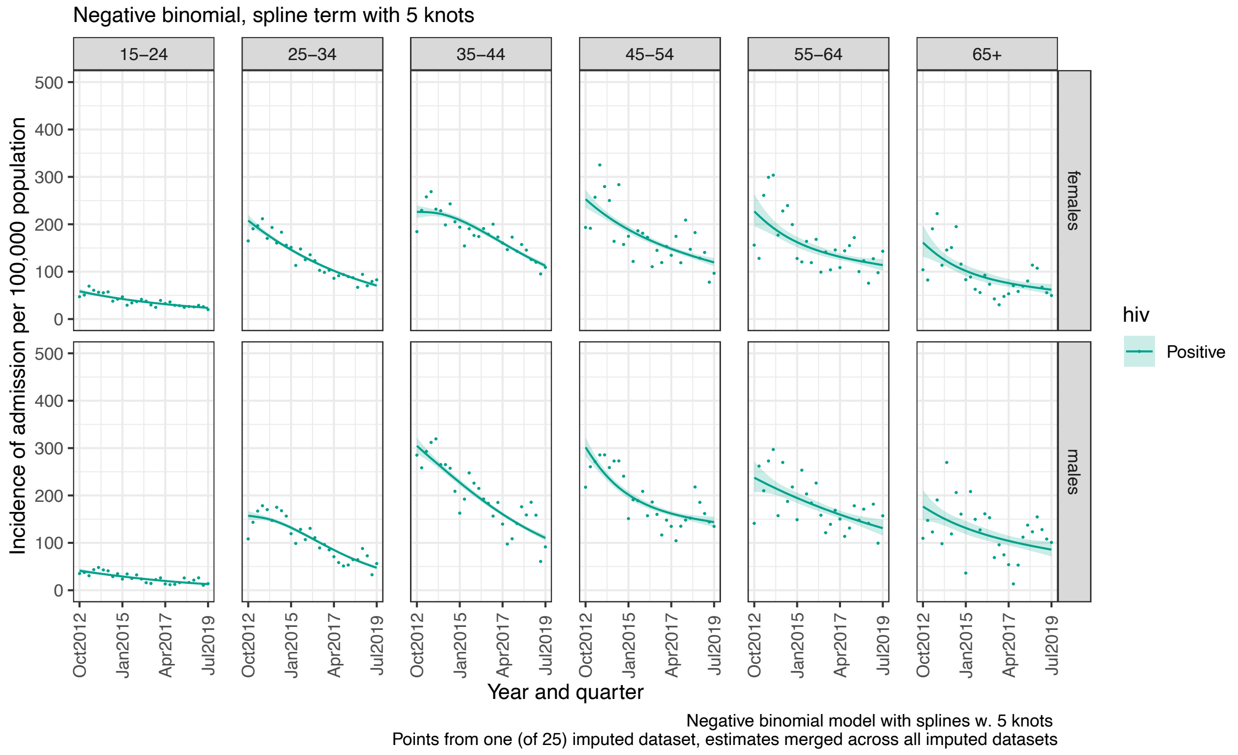

S. Figure 3C: Poisson distribution, 3 knot splines

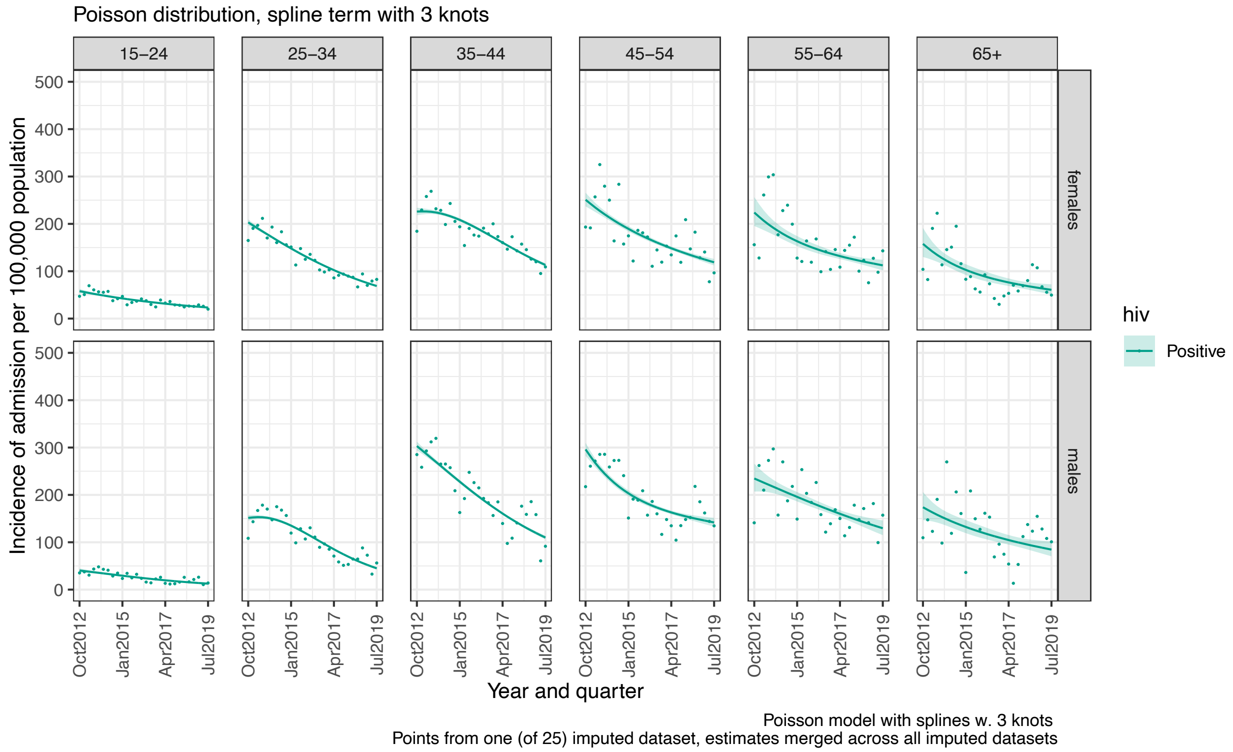


S. Figure 3D: Gamma distbution, no splines

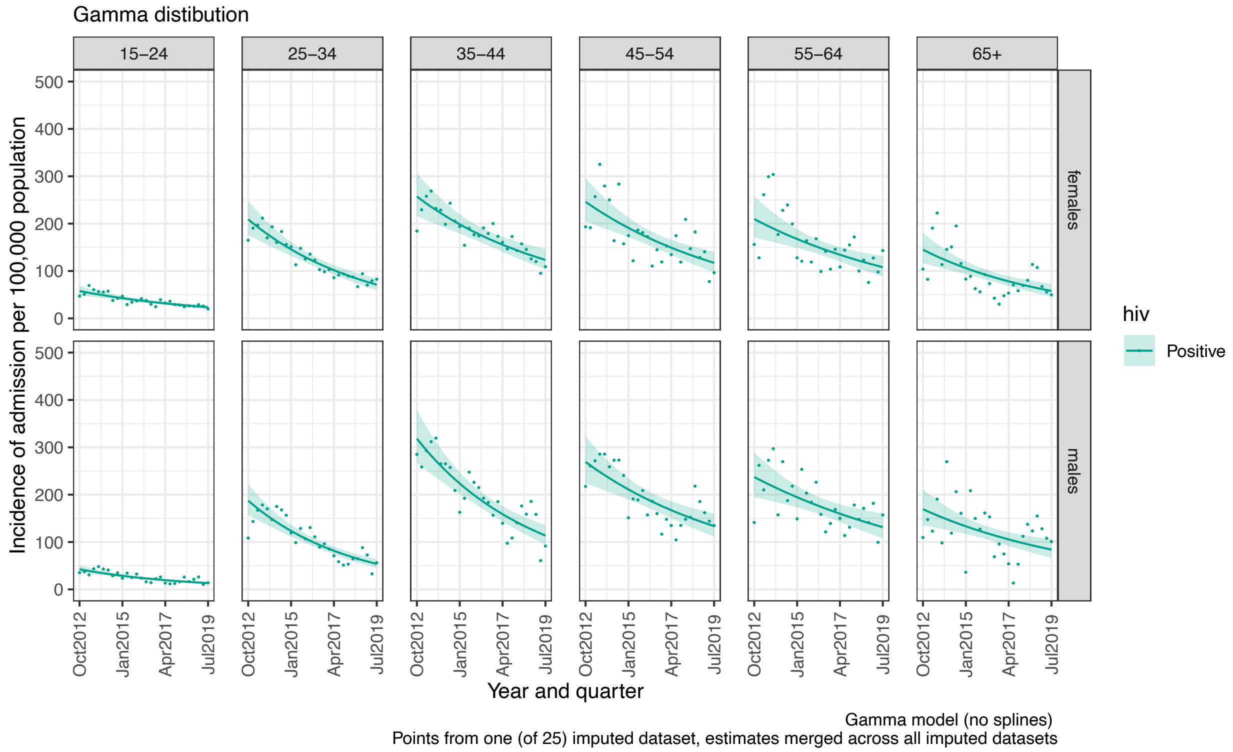

**Supplementary table 3: Death risk by age group and sex**

Table 3A: Crude Data

| hivart | | (Missing) (N=2687) | Died (N=6071) | Discharged alive (N=24056) |
|---|---|---|---|---|
| HIV negative | **ageg10** | | | |
| | 15-24 | 73 (4.1%) | 124 (7.0%) | 1575 (88.9%) |
| | 25-34 | 66 (4.2%) | 135 (8.6%) | 1366 (87.2%) |
| | 35-44 | 96 (8.5%) | 123 (10.9%) | 909 (80.6%) |
| | 45-54 | 79 (9.5%) | 104 (12.6%) | 645 (77.9%) |
| | 55-64 | 119 (11.1%) | 143 (13.4%) | 809 (75.5%) |
| | 65+ | 258 (12.6%) | 323 (15.8%) | 1463 (71.6%) |
| | **sex** | | | |
| | females | 403 (8.6%) | 448 (9.6%) | 3814 (81.8%) |
| | males | 288 (7.7%) | 504 (13.5%) | 2953 (78.9%) |
| HIV positive, ART status unknown | **ageg10** | | | |
| | 15-24 | 3 (15.8%) | 2 (10.5%) | 14 (73.7%) |
| | 25-34 | 22 (22.9%) | 18 (18.8%) | 56 (58.3%) |
| | 35-44 | 20 (19.6%) | 16 (15.7%) | 66 (64.7%) |
| | 45-54 | 3 (8.3%) | 6 (16.7%) | 27 (75.0%) |
| | 55-64 | 6 (30.0%) | 7 (35.0%) | 7 (35.0%) |
| | 65+ | 0 (0.0%) | 4 (36.4%) | 7 (63.6%) |
| | **sex** | | | |
| | females | 24 (20.2%) | 19 (16.0%) | 76 (63.9%) |
| | males | 30 (18.2%) | 34 (20.6%) | 101 (61.2%) |
| HIV positive, not on ART | **ageg10** | | | |
| | 15-24 | 15 (4.0%) | 73 (19.4%) | 288 (76.6%) |
| | 25-34 | 115 (10.8%) | 168 (15.8%) | 783 (73.5%) |
| | 35-44 | 110 (11.0%) | 179 (17.9%) | 712 (71.1%) |
| | 45-54 | 32 (8.5%) | 87 (23.2%) | 256 (68.3%) |
| | 55-64 | 11 (7.1%) | 31 (19.9%) | 114 (73.1%) |
| | 65+ | 9 (11.8%) | 20 (26.3%) | 47 (61.8%) |
| | **sex** | | | |
| | females | 123 (9.0%) | 195 (14.2%) | 1055 (76.8%) |
| | males | 169 (10.1%) | 363 (21.6%) | 1145 (68.3%) |
| HIV positive, on ART | **ageg10** | | | |
| | 15-24 | 138 (10.8%) | 184 (14.5%) | 951 (74.7%) |
| | 25-34 | 389 (9.8%) | 748 (18.8%) | 2838 (71.4%) |
| | 35-44 | 487 (10.6%) | 965 (20.9%) | 3160 (68.5%) |
| | 45-54 | 202 (10.2%) | 430 (21.7%) | 1346 (68.0%) |
| | 55-64 | 82 (9.6%) | 230 (26.8%) | 545 (63.6%) |
| | 65+ | 41 (10.8%) | 108 (28.5%) | 230 (60.7%) |
| | **sex** | | | |
| | females | 739 (10.2%) | 1210 (16.7%) | 5315 (73.2%) |
| | males | 600 (10.3%) | 1455 (25.0%) | 3755 (64.6%) |
| HIV status unknown | **ageg10** | | | |
| | 15-24 | 29 (2.1%) | 179 (13.1%) | 1160 (84.8%) |
| | 25-34 | 54 (3.2%) | 328 (19.3%) | 1318 (77.5%) |
| | 35-44 | 41 (3.1%) | 331 (25.1%) | 946 (71.8%) |
| | 45-54 | 42 (4.9%) | 223 (26.0%) | 592 (69.1%) |
| | 55-64 | 46 (5.3%) | 233 (27.0%) | 585 (67.7%) |
| | 65+ | 99 (5.2%) | 549 (29.1%) | 1241 (65.7%) |
| | **sex** | | | |

| hivart | | (Missing) (N=2687) | Died (N=6071) | Discharged alive (N=24056) |
|---|---|---|---|---|
| | females | 125 (3.9%) | 641 (20.1%) | 2431 (76.0%) |
| | males | 186 (3.9%) | 1202 (25.0%) | 3411 (71.1%) |

Table 3B: After imputation

Table 2: After imputation for missing data

| hivart | | Died (N=6656) | Discharged alive (N=26158) |
|---|---|---|---|
| HIV negative | **ageg10** | | |
| | 15-24 | 185 (7.5%) | 2280 (92.5%) |
| | 25-34 | 178 (9.2%) | 1763 (90.8%) |
| | 35-44 | 158 (11.7%) | 1193 (88.3%) |
| | 45-54 | 158 (15.6%) | 855 (84.4%) |
| | 55-64 | 245 (16.6%) | 1234 (83.4%) |
| | 65+ | 784 (22.5%) | 2707 (77.5%) |
| | **sex** | | |
| | females | 782 (12.8%) | 5321 (87.2%) |
| | males | 926 (16.4%) | 4711 (83.6%) |
| HIV positive by imputation, assume no ART for model | **ageg10** | | |
| | 15-24 | 135 (20.0%) | 540 (80.0%) |
| | 25-34 | 299 (22.5%) | 1027 (77.5%) |
| | 35-44 | 312 (28.5%) | 783 (71.5%) |
| | 45-54 | 190 (28.3%) | 482 (71.7%) |
| | 55-64 | 157 (34.4%) | 299 (65.6%) |
| | 65+ | 185 (41.9%) | 257 (58.1%) |
| | **sex** | | |
| | females | 408 (23.2%) | 1351 (76.8%) |
| | males | 870 (29.9%) | 2037 (70.1%) |
| HIV positive, not on ART | **ageg10** | | |
| | 15-24 | 75 (19.8%) | 303 (80.2%) |
| | 25-34 | 198 (18.1%) | 898 (81.9%) |
| | 35-44 | 213 (20.7%) | 816 (79.3%) |
| | 45-54 | 95 (25.0%) | 285 (75.0%) |
| | 55-64 | 33 (20.9%) | 125 (79.1%) |
| | 65+ | 25 (32.1%) | 53 (67.9%) |
| | **sex** | | |
| | females | 227 (16.2%) | 1174 (83.8%) |
| | males | 412 (24.0%) | 1306 (76.0%) |
| HIV positive, on ART | **ageg10** | | |
| | 15-24 | 211 (16.4%) | 1079 (83.6%) |
| | 25-34 | 863 (21.4%) | 3178 (78.6%) |
| | 35-44 | 1114 (23.8%) | 3572 (76.2%) |
| | 45-54 | 473 (23.5%) | 1536 (76.5%) |
| | 55-64 | 236 (27.0%) | 639 (73.0%) |
| | 65+ | 134 (34.5%) | 254 (65.5%) |
| | **sex** | | |
| | females | 1402 (19.1%) | 5953 (80.9%) |
| | males | 1629 (27.5%) | 4305 (72.5%) |

**Supplementary figure 4: Sensitivity analysis risk of death**

Complete case analysis, HIV and outcome unknown status removed

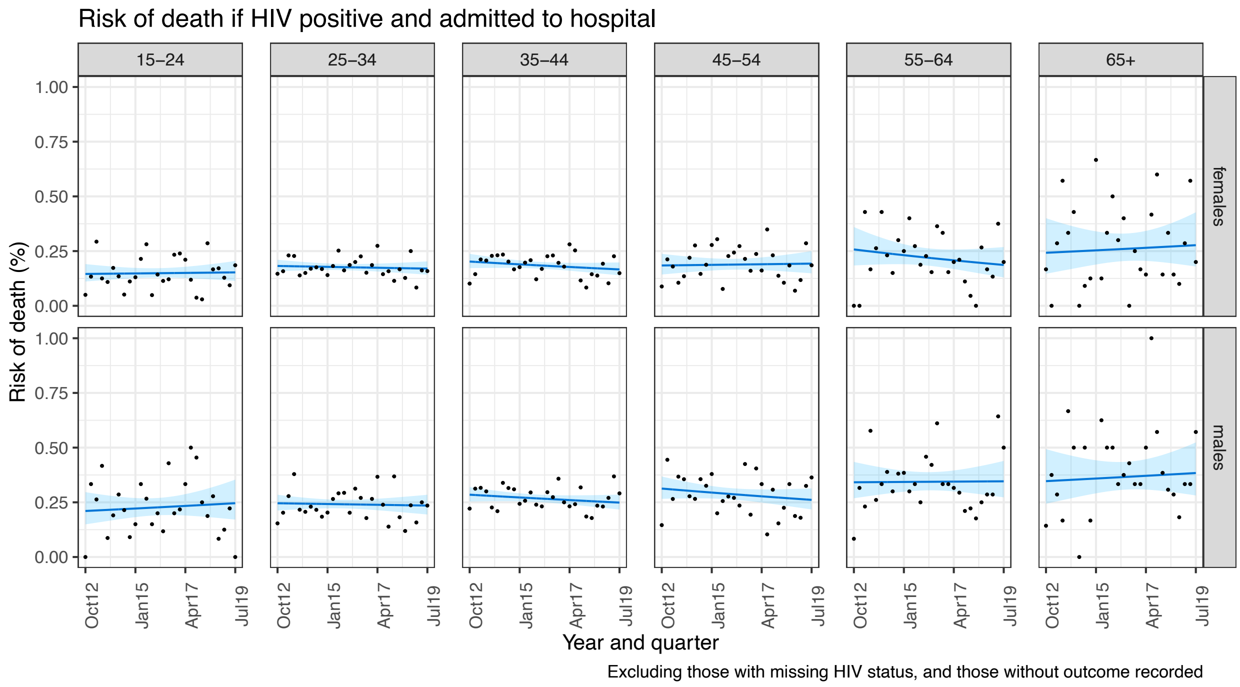


**Supplementary figure 5: Risk of death including ART as a covariate**

Plot model-predicted risk of death from model incorporating age group, sex, quarter-year and ART status

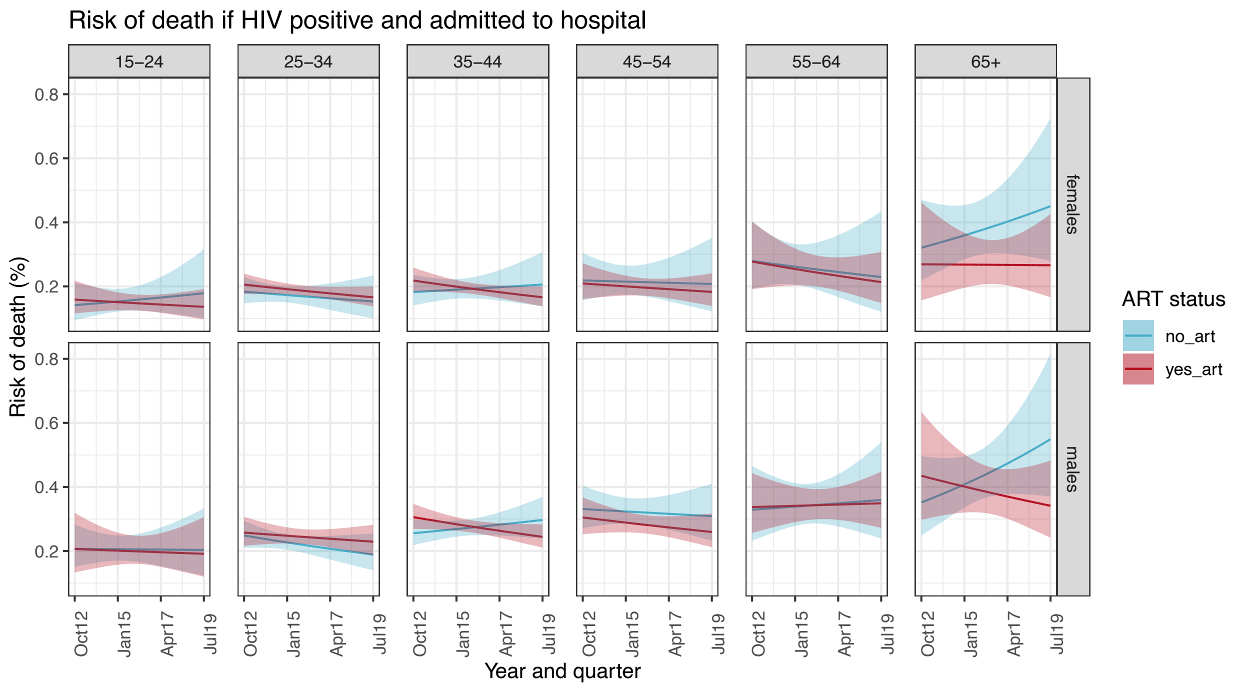

## Supplementary figure 6: Risk of death over time including HIV negative

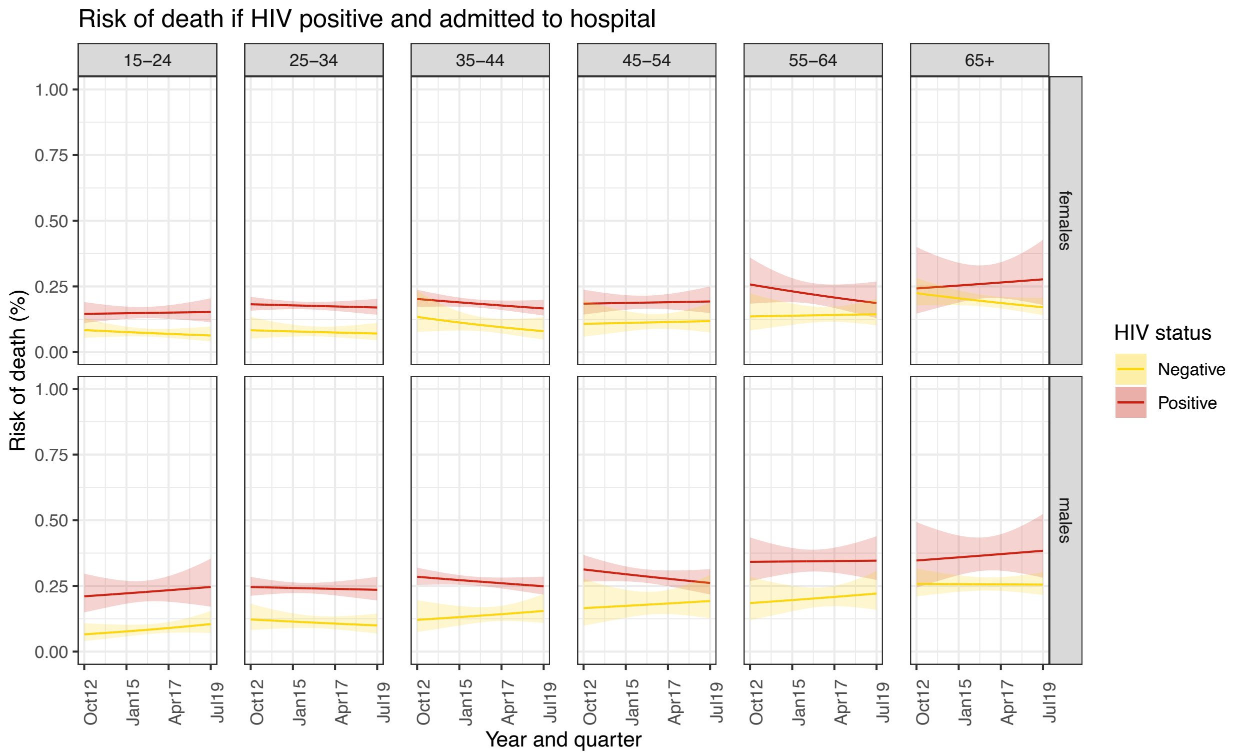

**Supplementary table 4: Trend in outcome by age and sex**

Using "emtrends" from "emmeans" package. In every age and sex group, confidence intervals for trend by quarter cross null effect.

| ageg10 | sex | q.trend | SE | df | asymp.LCL | asymp.UCL |
|---|---|---|---|---|---|---|
| 15-24 | females | 0.0004729613 | 0.0011636125 | Inf | -0.001807677 | 2.753600e-03 |
| 25-34 | females | -0.0010007736 | 0.0008266785 | Inf | -0.002621034 | 6.194864e-04 |
| 35-44 | females | -0.0017293607 | 0.0009121361 | Inf | -0.003517115 | 5.839319e-05 |
| 45-54 | females | -0.0011786709 | 0.0013566146 | Inf | -0.003837587 | 1.480245e-03 |
| 55-64 | females | -0.0039595377 | 0.0020453824 | Inf | -0.007968414 | 4.933821e-05 |
| 65+ | females | 0.0008105217 | 0.0027584539 | Inf | -0.004595949 | 6.216992e-03 |
| 15-24 | males | -0.0008936541 | 0.0016541745 | Inf | -0.004135777 | 2.348468e-03 |
| 25-34 | males | -0.0012515457 | 0.0010450936 | Inf | -0.003299892 | 7.968002e-04 |
| 35-44 | males | -0.0016206534 | 0.0009252838 | Inf | -0.003434176 | 1.928696e-04 |
| 45-54 | males | -0.0010379190 | 0.0013394894 | Inf | -0.003663270 | 1.587432e-03 |
| 55-64 | males | 0.0005027680 | 0.0020536922 | Inf | -0.003522395 | 4.527931e-03 |
| 65+ | males | 0.0015549194 | 0.0026720492 | Inf | -0.003682201 | 6.792040e-03 |

**Information about people who resided outside Blantyre**

5,511 people were excluded from analysis due to residence outside Blantyre. They had similar characteristics to people who lived in Blantyre.

HIV status of those inside and outside of Blantyre

| HIV | Blantyre | elsewhere |
|---|---|---|
| Unknown / Missing | 24.4% (7996) | 25.3% (1392) |
| Negative | 25.6% (8410) | 36.2% (1996) |
| Positive | 50.0% (16408) | 38.5% (2123) |

Age distribution of those inside and outside of Blantyre

| ageg10 | Blantyre | elsewhere |
|---|---|---|
| 15-24 | 14.7% (4808) | 17.1% (942) |
| 25-34 | 25.6% (8404) | 23.0% (1269) |
| 35-44 | 24.9% (8161) | 21.6% (1193) |
| 45-54 | 12.4% (4074) | 12.8% (704) |
| 55-64 | 9.0% (2968) | 9.9% (543) |
| 65+ | 13.4% (4399) | 15.6% (860) |

Sex distribution of those inside and outside of Blantyre

| sex | Blantyre | elsewhere |
|---|---|---|
| females | 50.6% (16618) | 53.9% (2971) |
| males | 49.4% (16196) | 46.1% (2540) |

Outcomes of those inside and outside of Blantyre

| outcome | Blantyre | elsewhere |
|---|---|---|
| 1. Survived to discharge | 73.3% (24056) | 71.6% (3946) |
| 2. Died in hospital | 18.5% (6071) | 23.3% (1283) |
| 3. Missing / unknown | 8.2% (2687) | 5.1% (282) |
